# A novel extradural nerve transfer technique by coaptation of C4 to C5 and C7 to C6 for treating isolated upper trunk avulsion of the brachial plexus

**DOI:** 10.7555/JBR.32.20180012

**Published:** 2018-05-12

**Authors:** Kaixiang Yang, Shaohua Zhang, Dawei Ge, Tao Sui, Hongtao Chen, Xiaojian Cao

**Affiliations:** Department of Orthopaedics, the First Affiliated Hospital of Nanjing Medical University, Nanjing, Jiangsu 210029, China.; Department of Orthopaedics, the First Affiliated Hospital of Nanjing Medical University, Nanjing, Jiangsu 210029, China.; Department of Orthopaedics, the First Affiliated Hospital of Nanjing Medical University, Nanjing, Jiangsu 210029, China.; Department of Orthopaedics, the First Affiliated Hospital of Nanjing Medical University, Nanjing, Jiangsu 210029, China.; Department of Orthopaedics, the First Affiliated Hospital of Nanjing Medical University, Nanjing, Jiangsu 210029, China.; Department of Orthopaedics, the First Affiliated Hospital of Nanjing Medical University, Nanjing, Jiangsu 210029, China.

**Keywords:** brachial plexus, nerve transfer, spinal nerve roots, extradural anastomosis, surgical, feasibility study

## Abstract

The study aimed to demonstrate the feasibility of an extradural nerve anastomosis technique for the restoration of a C5 and C6 avulsion of the brachial plexus. Nine fresh frozen human cadavers were used. The diameters, sizes, and locations of the extradural spinal nerve roots were observed. The lengths of the extradural spinal nerve roots and the distance between the neighboring nerve root outlets were measured and compared in the cervical segments. In the spinal canal, the ventral and dorsal roots were separated by the dura and arachnoid. The ventral and dorsal roots of C7 had sufficient lengths to anastomose those of C6. The ventral and dorsal of C4 had enough length to be transferred to those of C5, respectively. The feasibility of this extradural nerve anastomosis technique for restoring C5 and C6 avulsion of the brachial plexus in human cadavers was demonstrated in our anatomical study.

## Introduction

Brachial plexus root avulsion is a severe injury with a poor prognosis^[1]^. Upper trunk (C5 and C6) injury is a common form and accounts for over 30% of all brachial plexus injuries^[2]^. It causes the main symptoms of elbow flexion, external rotation and shoulder abduction dysfunction^[3]^.

Currently, nerve transfer within the plexus or surrounding the plexus is considered as the main treatment for brachial plexus root avulsion^[4^‒^5]^. The donor nerves for anastomosis include the accessory nerve^[6]^, phrenic nerve^[7]^, partial fascicles of the ulnar nerve^[8]^ and intercostal nerve^[9]^. Specifically, Yamada *et al*.^[10^‒^12]^ used C3-4 nerve roots and Gu *et al*.^[2]^ used the ipsilateral C7 nerve root to restore the upper trunk in brachial plexus injury. Both of them yielded satisfactory results and made a major breakthrough in the treatment of brachial plexus injury.

However, the above studies have some common limitations. Most of the nerve transfers were conducted out of the spinal canal. The donor nerves were postganglionic nerves containing motor and sensory neurofibers. If these fibers do not match accurately during neuroanastomosis, it may result in the decline of motor and sensory functions^[13]^. Furthermore, the above neuroanastomosis needs extra nerve grafts to meet the distance between recipients and donors. The clinical effects of extra nerve grafts are remarkably poorer than those of direct nerve suturing. Thus, in order to reduce the above limitations, an accurate, prompt and convenient technique needs to be developed.

Our previous study showed that the extradural ventral and dorsal roots can be successfully separated and anastomosed to treat bladder reinnervation after spinal cord injury^[14]^. On the basis of the previous research, our study aimed to demonstrate the feasibility of an extradural nerve anastomosis technique for treating upper trunk avulsion of the brachial plexus through an anatomical technique in human fresh frozen cadavers.

## Materials and methods

### Cadavers

Five male and four female fresh frozen cadavers (18 sides; mean age, 48 years old; range, 35 to 73 years) were obtained from the Department of Anatomy at Nanjing Medical University. Legal surrogates for cadaver donors signed an informed written consent form. The Ethics Committee of Nanjing Medical University approved this study. Specimens with kyphosis, lordosis, scoliosis, prior spinal surgery and intervertebral disc disease were excluded. 

### Dissection techniques

Gross dissection techniques were used to certify the feasibility of this modified extradural neuroanastomosis method. In each cadaver, dissection proceeded via the following approaches: 1) exposure of the C4 to T1 extradural nerve roots by means of limited laminectomy; 2) separation of the ventral and dorsal roots from the extradural nerve root by the microdissection technique; 3) transferring the C4 nerve root to C5 nerve root and C7 nerve root to C6 nerve root by the microscopic suture technique.

To assess whether the distance between the donor and recipient nerves is long enough so that the donor and recipient nerves can be matched, the length of extradural nerve roots and the distance between the neighboring nerve root outlets were measured. In detail, the length of the extradural nerve roots was considered as the distance between the nerve root outlets (i.e., its emergence from the spinal cord dura mater) and the center of the spinal dorsal root ganglion. A caliper was used to measure the above distances with a precision of 0.01 mm. The diameters of each nerve root were measured by a caliper (0.01 mm).

### Data analysis

Excel 2007 software (Microsoft, Seattle, WA, USA) was used to collect all data. The SPSS 22.0 software (SPSS, Chicago, IL, USA) was used for further statistical analysis, and the measurement of the pertinent distances and the diameters of the extradural nerve roots were determined by mean and standard deviation (SD). Student's *t*-test was used to determine statistical significance of differences among the means. A *P*-value of 0.05 was considered significant.

## Results

### Exposure of C4 to T1 extradural nerve roots

**Fig.1:**
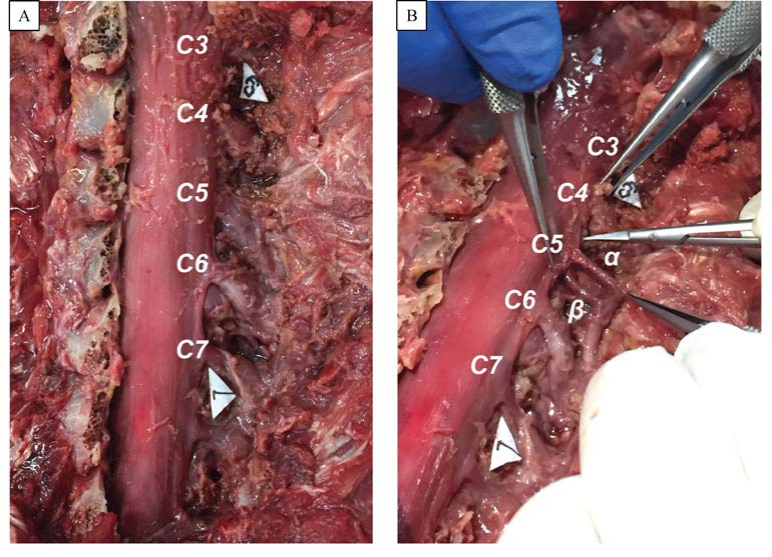
Overviews of the dural sac and extradural spinal nerve roots. A: The spinal nerve root from C3 to C7. B: Demonstration of the feasibility of dividing the ventral root from the dorsal root outside of the dural sac. α indicates the dorsal root, and β indicates the ventral root.

The exposure dissection involved a midline incision on the level of C4 to T1 segments. The soft tissues were completely removed until the spinous processes and the corresponding vertebra were reached. A laminectomy was performed at the level of C4 to T1 segments. Then, the extradural nerve roots and dural sac were exposed in the corresponding segments **(** ***Fig. 1A*****)**. After exposure of the extradural nerve roots, we found that each extradural nerve root descended from the nerve root outlet and the corresponding intertebral foramina. The dorsal root ganglionic enlargement was adjacent to the intertebral foramina.

### Separation of the ventral and dorsal roots in the C4-T1 segment

After the C4-T1 segment extradural nerve roots were exposed, an incision was made in the spinal dural mater at the location of the dorsal root ganglion with a microsurgical instrument. We found that both the ventral root and the dorsal root were individually surrounded by connective tissues, and the connective tissues of the ventral and dorsal roots were loose and easily separated. Subsequently, we carefully separated the connective tissues between the ventral and dorsal roots with microstructural scissors, and separated proximally to the spinal nerve roots outlet and distally to the junction of the ventral and dorsal roots (***Fig. 1B***). Then, we used the same method to separate the C4-T1 extradural spinal nerve roots.

### Transfer of the C4 nerve root to C5 nerve root and C7 nerve root to C6 nerve root

**Fig.2:**
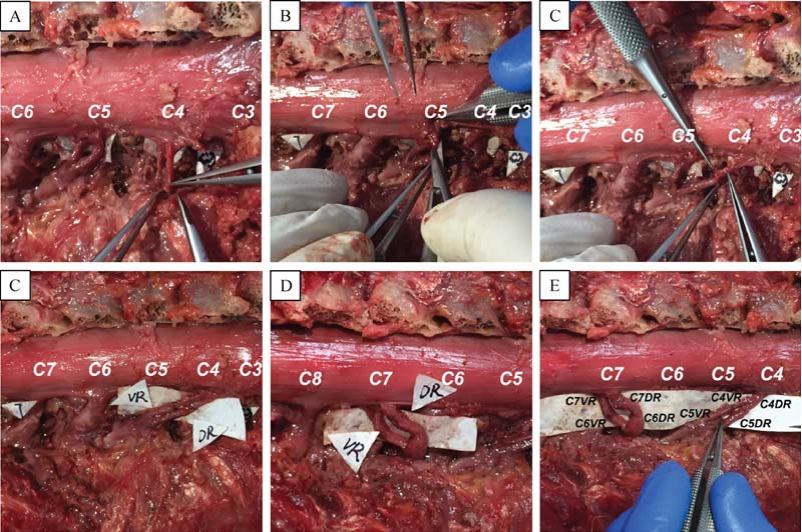
The operating process of the experimental extradural nerve root anastomosis (C4-C5; C6-C7). A: The ventral and dorsal roots of C4 were separated out of the dural sac and were cut close to the dorsal root ganglion. B: The ventral and dorsal roots of C5 were separated out of the dural sac and were cut near to the nerve root outlet. C: The C4 ventral root anastomosed with the C5 ventral root. D: The C4 dorsal root anastomosed with the C5 dorsal root. E: The ventral and dorsal roots of C6 anastomosed with those of C7, respectively. F: The overviews of the outcome of the extradural nerve root anastomosis technique, and all the matches were without tension. VR indicates ventral root, DR indicates dorsal root.

**Fig.3:**
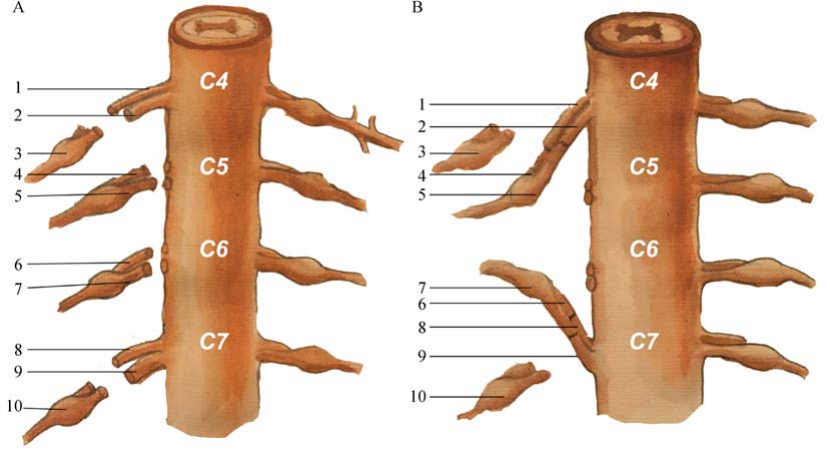
A schematic diagram of the experimental extradural nerve root anastomosis (C4‒C5; C6‒C7). A: the ventral and dorsal roots of C4 to C7 segments were cut off. B: the C4 and C7 nerve roots were anastomosed with C5 and C6 nerve roots, respectively. 1: the C4 ventral root; 2: the C4 dorsal root; 3: the C4 dorsal root ganglion; 4: the C5 ventral root: 5: the C5 dorsal root; 6: the C6 ventral root; 7: the C6 dorsal root; 8: the C7 ventral root; 9: the C7 dorsal root; 10: the C7 dorsal root ganglion.

When the ventral and dorsal roots from C4 to T1 segments were separated, the nerve roots of the C5 and C6 segments were severed near the dural sac, and the nerve roots of the C4 and C7 segments were severed near the junction of the ventral and dorsal roots. Subsequently, the distal end of the ventral and dorsal roots of the C4 segments and the proximal end of the ventral and dorsal roots of the C5 segments were anastomosed respectively. The distal end of the ventral and dorsal roots of the C7 segments and the proximal end of the ventral and dorsal roots of the C6 segments were anastomosed respectively. The surgical operation of the nerve root anastomosis is shown in ***Fig. 2***. The diagram of the anastomosis technique is shown in ***Fig. 3***.

**Fig.4:**
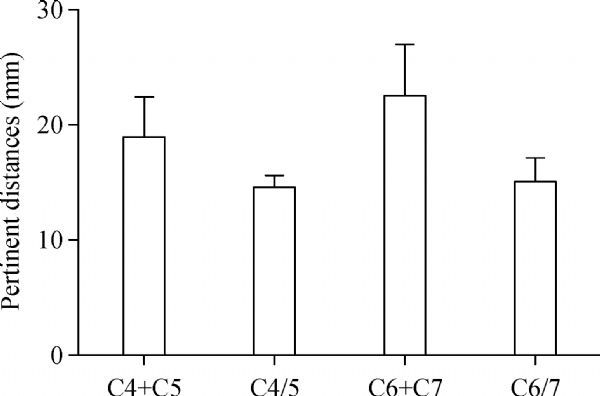
Bar graph shows the results of the pertinent distances referred in the study. C4 + C5 indicates the sum of the length of C4 nerve root and C5 nerve root. C6 + C7 indicates the sum of the length of C6 nerve root and C7 nerve root. C4/5 indicates distance of the neighboring nerve root outlets between C4 and C5. C6/7 indicates distance of the neighboring nerve root outlets between C6 and C7.

**Fig.5:**
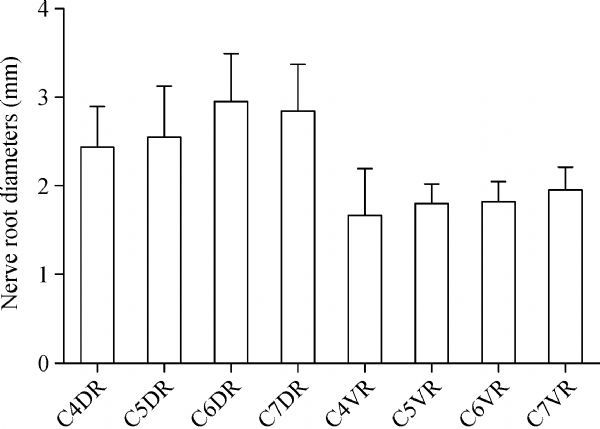
Bar graph shows the results of the diameters of each nerve root from C4 to C7. VR indicates ventral root, DR indicates dorsal root.

In addition, we measured the distance between the nerve root outlet of the C4 segments and the junction of the ventral and dorsal roots of the C5 segments (d45), and the distance between the nerve root outlet of the C7 segments and the junction of the ventral and dorsal roots of the C6 segments (d67). Comparing the distance relationship of the d45 and the sum of the C4 extradural nerve root length and the C5 extradural nerve root length, we found that the C4 ventral and dorsal roots had sufficient length to tension-free anastomose the C5 ventral and dorsal roots, respectively. Comparing the distance relationship of the d67 and the sum of the C6 extradural nerve root length and the C7 extradural nerve root length, we found that the C6 ventral and dorsal roots had sufficient length to tension-free anastomose the C7 ventral and dorsal roots, respectively (***Fig. 4***). Comparing the diameters of the donor and recipient nerves, we found that in both ventral and dorsal roots, there was no significant difference between the diameters of C4 and C5, and between those of C6 and C7 (*P*>0.05, ***Fig. 5***). Thus, these results of the pertinent distances and the diameters coincided with our experimental anastomosis results.

## Discussion

Our study demonstrated the applied anatomy of the extradural spinal nerve roots of the cervical segments. We found and proved the possibility of a novel extradural nerve transfer technique for treating C5 and C6 avulsion of the brachial plexus.

The choice of the donor nerves for treating C5 and C6 avulsion has become a focus in current research^[15]^. Previous studies suggested that no functional impairment occurred in patients with isolated C7 injury^[16^‒^17]^. The healthy-side C7 nerve root transfer has been proved as an efficacious and safe surgery for treatment of total root avulsion of the brachial plexus^[18^‒^20]^. In some clinical researches, the ipsilateral C7 nerve root was also used as the donor nerve for repair of the upper trunk of the brachial plexus and the results showed that the treatment caused no neurophysiological, electromyographic, or functional disturbance^[21^‒^22]^. Therefore, it is safe and efficacious to choose the ipsilateral C7 nerve root as the donor nerve.

What’s more, the axon number of donor nerves was one of the key factors determining the efficacy of nerve transfer, and the smallest proportion of the donor nerve axon number that could maintain the target organ function was about 40%^[23]^. In one of our previous studies, the axon number of nerve roots from C1 to S5 was measured and we found that C7 nerve root had a sufficient number of axons for functional recovery when being transferred to the C5 or C6 nerve root^[24]^. Therefore, the ipsilateral C7 nerve root was used as one of the donor nerves for treating the C5 and C6 nerve root avulsion in our study. However, in the process of neurorrhaphy, we found that the extradural nerve root is long enough for neuroanastomosis between C7 and C6, but is not long enough for neuroanastomosis between C7 and C5. Thus, extra bridging nerves or extra donor nerves should be applied in the study.

In the previous study, the anterior rami of C3 and C4 were applied as donors to repair the upper trunk of the brachial plexus root avulsion with an extracted nerve graft^[10^‒^12]^. Since the main branch of the C4 nerve root is the phrenic nerve, phrenic nerve transfer has been applied in many centers and achieved satisfactory clinical efficacy without causing any remarkable effect to pulmonary function^[25^‒^27]^. Therefore, according to the above studies, the C4 and C7 nerve roots were used as the donor nerves to treat C5 and C6 avulsion in our study.

The current nerve anastomosis technique for treating the C5 and C6 avulsion of the brachial plexus included the transfer of phrenic nerve, accessory nerve, intercostal nerves, and partial fascicles of the ulnar nerve^[6^‒^9]^. All of the anastomosis was conducted out of the spinal canal and all of the donor and recipient nerves were peripheral nerves. Since the ventral nerve root (motor nerve) and the dorsal nerve root (sensory nerve) were intersected behind the dorsal root ganglion, the anastomosis of the postganglionic nerves would cause mismatches between motor and sensory fibers of donors and recipients.

To avoid or reduce the aforementioned defects, our study modified the surgical procedure by performing the extradural spinal nerve roots anastomosis instead of current nerve anastomosis techniques. In the process of the dissection, we found that the ventral and dorsal roots were easy to separate. According to the anatomical features, the ventral root was accurately transferred to the ventral root, and the dorsal root was accurately transferred to the dorsal root. In other words, the motor and sensory nerve fibers of the donor nerves were accurately transferred to those of the recipient nerves, respectively. Thus, the extradural nerve root anastomosis technique has the advantages of a simple surgical procedure and can promote the nerve regeneration remarkably.

Tensional anastomosis can influence neurological function restoration^[28]^. Thus, the sufficient lengths of the donor and the recipient nerve are essential for nerve anastomosis. In our study, we found that the C4 ventral and dorsal root had enough length to be transferred, without tension, to the C5 ventral and dorsal root separately. The length of extradural nerve roots is also enough for nerve anastomosis between C6 and C7. These results were also corroborated by our experimental anastomosis.

Different diameters of donor and recipient nerves might lead to difficulty in anastomosis and influence functional recovery. Thus, choosing donors with similar diameters of recipients is essential^[24]^. Our measurements showed no remarkable difference between the ventral root diameters of C4 and C5 and between the dorsal root diameters of C4 and C5 (*P*>0.05). Similarly, no significant differences were found between the ventral root diameters of C6 and C7 and between the dorsal root diameters of C6 and C7 (*P*>0.05). Thus, in our study, the diameters of the donor nerves (C4 and C7) were similar to those of the recipients’ nerves (C5 and C6).

However, there are some limitations in this novel extradural nerve root anastomosis technique, including potential injury to the dorsal root ganglion and difficulties in surgical dissection. Furthermore, because of the limited cadaver number, the anatomical data of the extradural spinal nerve roots are for reference only, and further research is required.

In conclusion, this study provides an anatomical basis for a novel extradural nerve root anastomosis technique to treat C5 and C6 avulsion of the brachial plexus. By means of this technique, the C4 ventral root anastomosed the C5 ventral and dorsal root, respectively, and the C7 ventral and dorsal root were transferred to the C6 ventral and dorsal root. Accurate anastomosis (i.e., motor-to-motor and sensory-to-sensory nerves) and no extra nerve grafting are the greatest advantages of this technique. This novel technique demonstrates great potential for clinical application in patients with a C5 and C6 avulsion of the brachial plexus, and we will observe the clinical effects of this technique in a future study.
